# Development of an Efficient G‐Quadruplex‐Stabilised Thrombin‐Binding Aptamer Containing a Three‐Carbon Spacer Molecule

**DOI:** 10.1002/cbic.201600654

**Published:** 2017-03-15

**Authors:** Lukas J. Aaldering, Vasanthanathan Poongavanam, Niels Langkjær, N. Arul Murugan, Per Trolle Jørgensen, Jesper Wengel, Rakesh N. Veedu

**Affiliations:** ^1^ Nucleic Acid CenterDepartment of PhysicsChemistry and PharmacyUniversity of Southern DenmarkCampusvej 555230Odense MDenmark; ^2^ Institute for Plant Biology and BiotechnologyWestphalian Wilhelms University MünsterSchlossgarten 348149MünsterGermany; ^3^ Division of Theoretical Chemistry and BiologySchool of BiotechnologyRoyal Institute of Technology (KTH)10691StockholmSweden; ^4^ Centre for Comparative GenomicsMurdoch UniversityMurdochPerth6150Australia; ^5^ Western Australian Neuroscience Research InstituteMurdochPerth6150Australia; ^6^ School of Chemistry and Molecular BiosciencesThe University of QueenslandSt. LuciaBrisbane4072Australia

**Keywords:** aptamers, G-quadruplex, modified DNA, thrombin, unlocked nucleic acid

## Abstract

The thrombin‐binding aptamer (TBA), which shows anticoagulant properties, is one of the most studied G‐quadruplex‐forming aptamers. In this study, we investigated the impact of different chemical modifications such as a three‐carbon spacer (spacer‐C_3_), unlocked nucleic acid (UNA) and 3′‐amino‐modified UNA (amino‐UNA) on the structural dynamics and stability of TBA. All three modifications were incorporated at three different loop positions (T3, T7, T12) of the TBA G‐quadruplex structure to result in a series of TBA variants and their stability was studied by thermal denaturation; folding was studied by circular dichroism spectroscopy and thrombin clotting time. The results showed that spacer‐C_3_ introduction at the T7 loop position (TBA‐SP7) significantly improved stability and thrombin clotting time while maintaining a similar binding affinity as TBA to thrombin. Detailed molecular modelling experiments provided novel insights into the experimental observations, further supporting the efficacy of TBA‐SP7. The results of this study could provide valuable information for future designs of TBA analogues with superior thrombin inhibition properties.

## Introduction

**Scheme 1:**
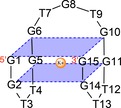
G‐quadruplex structure of the thrombin‐binding aptamer.

Oligonucleotide therapies have great potential and have attracted significant interest in recent years. Aptamers are a class of short, single‐stranded oligonucleotide sequences that are able to bind target molecules with high affinity and specificity because of their ability to adopt three‐dimensional structures. Aptamers, often termed chemical antibodies, are generally developed by an in vitro selection process called systematic evolution of ligands by exponential enrichment (SELEX).^1^, ^2^, ^3^ The developed full‐length aptamers can be chemically fabricated and truncated by using predicted secondary structures to further improve their biophysical properties.^4^ The thrombin‐binding aptamer (TBA) is one of the most studied G‐quadruplex forming aptamers.^5^ It is a 15‐mer G‐rich DNA oligonucleotide (5′‐GGTTG GTGTG GTTGG‐3′, Scheme 1) that inhibits the formation of fibrin clots by binding to thrombin,5c which makes TBA a therapeutically relevant anticoagulant. However, TBA did not meet clinical expectations during trial stages due to suboptimal dosing profiles; consequently, development beyond phase I clinical studies was not initiated.^6^ Since then, the investigation and improvement of TBA have been continued.^5^

X‐ray crystallography and NMR spectroscopy studies revealed that TBA forms a chair‐shaped antiparallel G‐quadruplex.^7^, ^8^, ^9^ The structure consists of two stacked G‐tetrads connected through three edgewise loops: a top central TGT and two bottom TT loops (Scheme 1). With regard to the structure and interaction with thrombin, a number of investigations have shown that the loop regions of TBA are crucial for thrombin interaction and G‐quadruplex formation.^10^, ^11^ Previous structure–activity relationship (SAR) studies revealed that loop residues T4, T9, T13 and G8 are critical to preserve the G‐quadruplex structure, and that T3, T7 and T12 are more flexible moieties that are particularly involved in thrombin inhibition.^12^, ^13^, ^14^, ^15^ Krauss et al. suggested that the TT loops form a pincer‐like structure that binds the protruding region of thrombin exosite I.^11^ In addition, it was further suggested that the stability and rigidity of TBA is important for the interaction between the aptamer and exosite I.^16^ A large number of structural modifications have been tested to improve the activity and stability of TBA, often resulting in decreased stability of TBA compared to unmodified TBA.^12^, ^13^, ^14^, ^17^, ^18^ However, in the last year, several studies succeeded in improving upon TBA. Pasternak et al. reported that the systematic introduction of a single UNA‐U nucleotide at position T3, T7 or T12 increased the thermodynamic stability of TBA, and the presence of unlocked nucleic acid (UNA)‐U at position 7 showed high potency with regard to inhibition of fibrin clot formation.^14^ Borbone and colleagues reported similar results by modifying TBA at position T7 with an acyclic pyrimidine analogue.^13^ In two recent studies, Virgilio et al. was able to improve the functionality of TBA. In the first study, they introduced 5′‐fluoro‐2′‐deoxyuridine residues to positions T4 and T13, achieving remarkable improvements in melting temperatures and anticoagulant activity.^19^ In the second study, they added an extra residue at the 3′‐end or at both ends of the original TBA sequence, linked through 3′–3′ or 5′–5′ phosphodiester bonds, resulting in strong improvements in the thermal stability of TBA.^20^

**Scheme 2:**
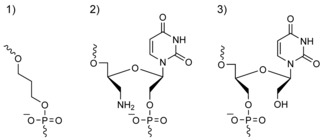
Structure of the three monomer modifications used in this study: 1) spacer‐C_3_ (X); 2) amino‐UNA (aU); and 3) UNA (uU).

Herein, we systematically investigated the effect of three different chemical modifications—3′‐amino‐modified UNA (amino‐UNA), unlocked nucleic acid (UNA) and a three‐carbon spacer (spacer‐C_3_; Scheme 2)—at loop positions T3, T7 and T12, with the goal of improving the binding interactions with thrombin. In the amino‐UNA monomer included in this study, the 3′‐OH group was replaced with a NH_2_ group; thus, the linkage to the next nucleotide became a 5′–2′ internucleotide phosphate linkage. A small library of modified TBA sequences was synthesised (Table 1) by varying three substitutions of residues T3, T7 and T12 with amino‐UNA, UNA and spacer‐C_3_ modifications (Table 1). The sequences were tested for quadruplex folding by circular dichroism (CD) spectroscopy, thermal stability of the formed structure by melting temperature (*T*_m_) analysis, a thrombin clotting time assay and thrombin binding affinity analysis of the most potent aptamer candidates by biolayer interferometry.

**Table 1:** Primary library of TBA derivatives.

Modification/sequence name	Sequence
TBA (reference)	5′‐GGTTGGTGTGGTTGG
aUNA (aU)/(TBA‐A3)	5′‐GG**aU**TGGTGTGGTTGG
aUNA (aU)/(TBA‐A7)	5′‐GGTTGG**aU**GTGGTTGG
aUNA (aU)/(TBA‐A12)	5′‐GGTTGGTGTGG**aU**TGG
spacer‐C_3_ (X)/(TBA‐SP3)	5′‐GG**X**TGGTGTGGTTGG
spacer‐C_3_ (X)/(TBA‐SP7)	5′‐GGTTGG**X**GTGGTTGG
spacer‐C_3_ (X)/(TBA‐SP12)	5′‐GGTTGGTGTGG**X**TGG
uUNA (uU)/(TBA‐U3)	5′‐GG**uU**TGGTGTGGTTGG
uUNA (uU)/(TBA‐U7)	5′‐GGTTGG**uU**GTGGTTGG
uUNA (uU)/(TBA‐U12)	5′‐GGTTGGTGTGG**uU**TGG

Modifications are shown in bold and underlined. aU: amino‐UNA, X: spacer‐C_3_ and uU: UNA.

## Results

### Physical properties and structural characterisation of modified TBA sequences

**Figure 1:**
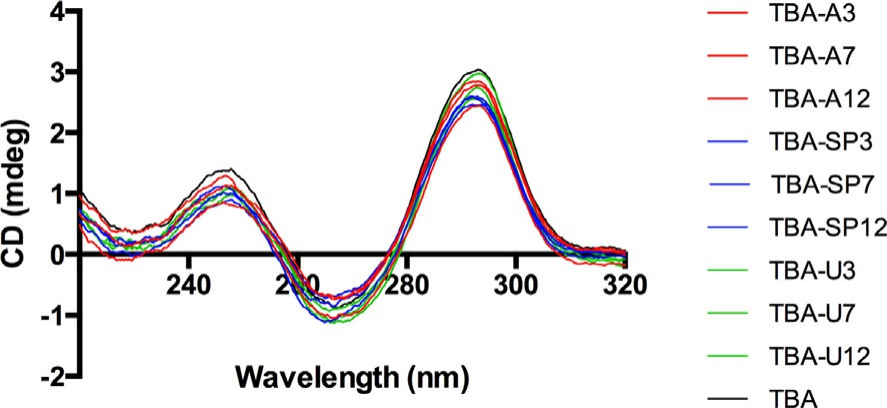
CD spectra of TBA and TBA derivatives.

Modified TBA sequences were characterised by CD spectroscopy to understand the effects of spacer‐C_3_, amino‐UNA and UNA modifications on the overall G‐quadruplex structure of TBA (Figure 1). All spectra showed the characteristic TBA absorbance^7^—two maxima at ≈240 and ≈295 nm and a minimum at ≈265 nm—thus suggesting the formation of antiparallel G‐quadruplex structures by the modification of TBA loops with spacer‐C_3_, amino‐UNA and UNA.

Next, the thermal stability of the quadruplexes formed by the TBA derivatives were analysed by UV melting studies (Figure S1 in the Supporting Information). The results showed a noteworthy increase (+≈6 °C) in thermal stability for substitutions with a spacer‐C_3_ (TBA‐SP3, TBA‐SP7 and TBA‐SP12), but the TBA variants with UNA monomers (TBA‐U3, TBA‐U7 and TBA‐U12) showed an increase of +2 to +5 °C (Table 2 and Figure S1). However, the introduction of amino‐UNA to the loops (TBA‐A3, TBA‐A7 and TBA‐A12) decreased the thermal stability in comparison with the unmodified TBA (Table 2).

**Table 2:** *T*_m_ values and thrombin clotting times of TBA and modified TBA sequences.

Oligonucleotide	*T*_m_	Δ*T*_m_	Thrombin	Δ Thrombin
	[°C]	[°C]	clotting	clotting
			time [s]	time [s]
thrombin	–	–	29±6	–
TBA	49±0	–	87±11	–
TBA‐A3	47±1	−2	55±8	−32
TBA‐A7	42±0	−7	51±3	−36
TBA‐A12	45±1	−4	45±8	−42
TBA‐SP3	55±0	+6	41±7	−46
TBA‐SP7	55±0	±6	168±39	±81
TBA‐SP12	55±1	+6	47±10	−40
TBA‐U3	51±0	+2	36±5	−51
TBA‐U7	54±0	+5	51±11	−36
TBA‐U12	51±0	+2	37±4	−50

### Biological effect of the modified TBA sequences

To investigate the ability of the modified TBA sequences to inhibit the enzymatic activity of thrombin and to evaluate the impact of quadruplex thermal stability on biological potency, the sequences were tested in a thrombin clotting time assay where the change in absorption, due to thrombin‐induced clotting of fibrinogen in phosphate‐buffered saline (PBS), was spectrophotometrically measured at 380 nm. The time needed to clot 50 % of the available fibrinogen (“clotting time”) was then determined. The clotting time without aptamer interference was (29±6) s. The majority of the modified TBA sequences showed decreased inhibitory efficiency compared to unmodified TBA, but TBA‐SP7 showed remarkably enhanced clotting time (Table 2). To validate this remarkable difference of TBA‐SP7 in the clotting time assay, further investigation of the thrombin aptamer binding affinity was investigated by biolayer interferometry using the BLITz instrument platform (ForteBio). However, TBA and TBA‐SP7 exhibited similar affinities (1665 nm for TBA‐SP7 and 1733 nm for TBA; Figure S2).

### Computational analysis

**Figure 2:**
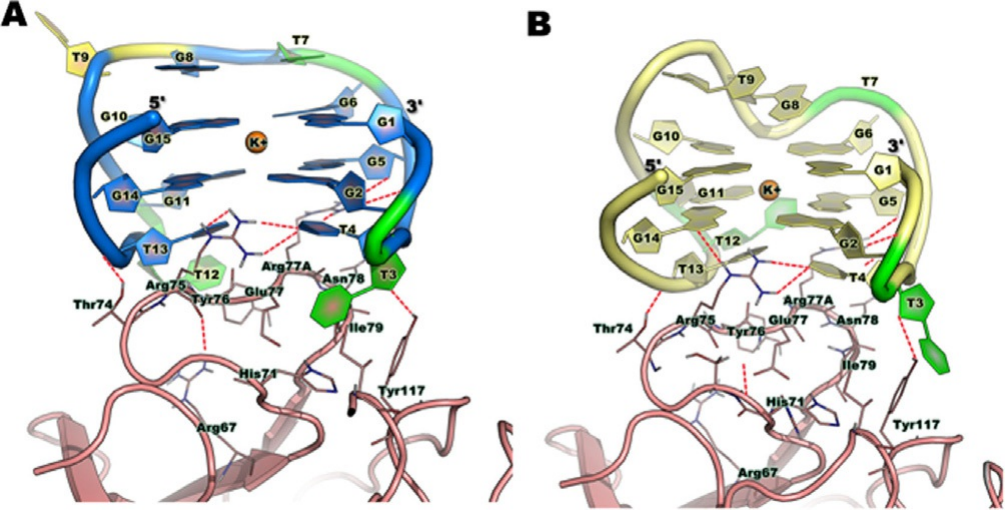
Comparison of molecular interactions of A) TBA and B) TBA‐SP7 with thrombin (maroon). The polar interactions between TBA and thrombin are shown, and important residues that are involved in TBA binding are highlighted.

The effect of the introduction of different monomers was further investigated by using the stochastic dynamics (SD) simulations. The model for TBA (Figure 2 A) shows guanine and thymine base stacking at positions 7 and 8 in the TGT loop, whereas the nucleobase of the thymidine residue at position 9 is not involved in the stacking and is directed upwards and away from the G‐quadruplex core. T3 and T12 adopt a pincer‐like structure.

It has been reported that the TT loops (T3 and T12) constitute the thrombin‐binding motifs of TBA^11^ and that these pincer‐like loops bind the protruding region of thrombin exosite I. Therefore, we focused on the structural changes in the pincer‐like orientation of T3 and T12, as well as on T9 of the TGT loop. On the other hand, TBA‐SP7 (Figure 2 B) showed strong binding interactions at thrombin exosite I as compared to the TBA; this is mainly due to the fact that there are more interactions with TBA‐SP7, especially at G14, in addition to G2, T3 and T4, which are common to both TBA and TBA‐SP7 analogues. Noticeably, there is a unique π–π interaction between the residue Tyr118 and T3 of TBA‐SP7. These interactions strongly stabilise the TBA‐SP7 conformation compared to the other TBA analogues designed in this study. For a better overview, a molecular model was used to examine conformational changes of TBA upon incorporation of spacer‐C_3_, amino‐UNA and UNA at positions T3, T7 and T12. Conformational changes of various tested aptamers binding to TBA are provided in the Supporting Information (Figure S2).

Table 3 displays the conformational changes and the results for thrombin clotting time activity (CT_50_; the time needed to clot 50 % of the available fibrinogen). The introduction of amino‐UNA in TBA always changed the orientation of the T9 nucleobase from an upward direction to an orientation more towards the side and away from the guanine tetrad. Additionally, the pincer‐like structure of the TT loops was disrupted for the amino‐UNA introduction at T3 and T12. Spacer‐C_3_ incorporation at T3 and T12 (TBA‐SP3 and TBA‐SP12) resulted in the loss of the pincer‐like structure and the wild‐type orientation of T9. However, in the case of TBA‐SP7, only the organisation of the TGT loop was affected. Similar to the data obtained for the spacer‐C_3_ variants, UNA variants TBA‐U3 and TBA‐U12 also showed the loss of the T3, T9 and T12 orientations. Interestingly, the structure of TBA‐U7 was very similar to the unmodified TBA, although not identical.

**Table 3:** Structural impact of substitution, ordered by site of modification, including orientation of T3, T7 and T12 and the impact on anti‐thrombin function compared to normal TBA.

Site	Chemistry	Orientation			CT_50_
		T3	T9	T12	
n.a.	unmodified	pincer‐like	upwards	pincer‐like	/
	TBA				
T3	aUNA	pincer‐like	sideways	interacting	–
				with G4	
	spacer‐C_3_	nil	sideways	interacting	—
				with G4	
	uUNA	interacting	sideways	interacting	—
		with G4		with G4	
T7	aUNA	pincer‐like	sideways	pincer‐like	–
	spacer‐C_3_	pincer‐like	interacting	pincer‐like	+++
			with G4		
	uUNA	pincer‐like	upwards	pincer‐like	–
T12	aUNA	downwards	sideways	interacting	–
				with G4	
	spacer‐C_3_	interacting	sideways	nil	–
		with G4			
	uUNA	interacting	sideways	interacting	—
		with G4		with G4	

A reduction of up to 50 % of anti‐thrombin activity (CT_50_) is indicated by (–), and reduction up to 75 % is marked (—); a significant increase is denoted by (+++).

All TBA derivatives shown in Table 1 were used for the prime molecular mechanics–generalised Born surface area (MM‐GBSA)‐based binding affinity calculations. CT_50_ values were compared with binding energies obtained from the MM‐GBSA calculations. The summary of the free energies obtained for each complex is provided in Table S1, which shows all the energy terms of Equation (1) together with the experimentally measured activities, as well as the predicted binding free energies (Δ*G*_bind_). TBA‐SP7, which had high potency (CT_50_=168 s) among the compounds tested, was correctly predicted as a highly stable compound by the MM‐GBSA method (−224.96 kcal mol^−1^). The coulomb energy for all of the compounds, obtained by the MM‐GBSA method, is highly favourable compared to the overall binding energy; however, significantly high solvation energy (*E*_solvation_) compensates for the electrostatic contribution to the overall binding process. As a result, the van der Waals (vdW) energy dominates the overall binding affinity for all the compounds. It is important to emphasise that the covalent binding energy (*E*_covalent_) contribution to the overall binding affinity of each complex is either negligible or unfavourable, except for the TBA‐SP7 complex. In the case of the TBA‐SP7–thrombin complex, the covalent binding energy contribution is remarkably low (−165.4 kcal mol^−1^), compared to rest of the compounds in this series, and points to this contribution as key to making TBA‐SP7 the most thermostable compound among those tested with respect to thrombin binding (Figure S3). The covalent binding energy was calculated from the energy difference between the thrombin‐bound and free conformations of aptamer and essentially refers to the contributions due to changes in intramolecular structure of aptamer upon binding to thrombin. In the case of TBA‐SP7, the covalent binding energy was as much as −165.4 kcal mol^−1^ and stabilises the thrombin:aptamer association to larger extent. The energy of the aptamer was relatively low when it was bound to thrombin compared to the conformation of aptamer existing in solvent (comparison of *E*_covalent_ and Δ*G*_covalent_ is provided in Figure S4 A in the Supporting Information). We further analysed the data in order to understand the covalent energy contribution and found no correlation (*R* ^2^=0.03) when Δ*G*_covalent_ was excluded from Δ*G*_bind_ in the dataset. This effect was mainly due to the TBA‐SP7 construct, which behaved as an outlier; however, when TBA‐SP7 and TBA were excluded from the dataset, the correlation improved to *R* ^2^=0.82 (Figure S4 B). This analysis clearly indicated that the high stability of TBA‐SP7 was mainly due to the Δ*G*_covalent_ energy, which also influenced the overall binding process to thrombin.

**Figure 3:**
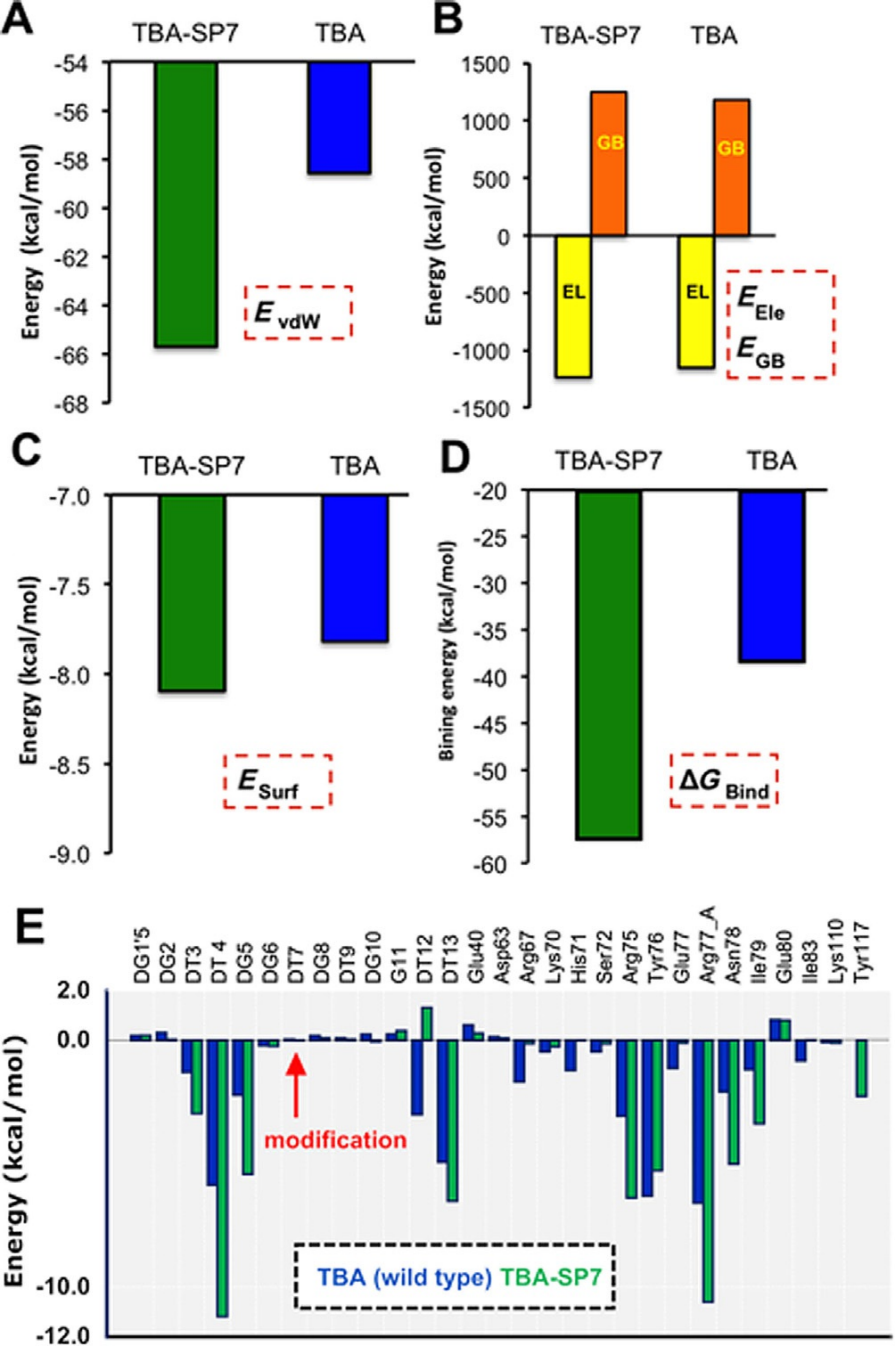
Comparison of different energy contributions for TBA and TPA‐SP7 thrombin binding from extensive MD simulation‐based MM‐GBSA calculation: A) van der Waals energy, B) electrostatic (Ele) energy and solvation generalized Born (GB), C) surface area energy—nonpolar energy, D) binding energy and E) decomposition of binding energy. All energy components were extracted from the differences (average) of Δ*G*_complex_−Δ*G*_thrombin_− Δ*G*_aptamer_. Some residues were removed from the plot (E), due to an insignificant energy contribution (<0.1 kcal mol^−1^).

In addition to prime MM‐GBSA calculations, an additional extensive molecular dynamics simulation with explicit solvation was performed for the highly active construct, TBA‐SP7, in order to compare the binding mode with TBA (wild‐type) with respect to binding free energies. Based on the 15 ns simulations, the relative binding energies of TBA and TBA‐SP7 in thrombin were estimated. From Figure 3, it is clear that TBA‐SP7 binds (Δ*G*_bind_=−57.19±0.2) relatively more strongly than TBA (Δ*G*_bind_=−38.26±0.3) in the thrombin binding site. An individual energy contributions plot also reveals that nonpolar contribution dominates the binding, as TBA‐SP7 showed significantly higher *E*_vdW_ and *E*_surf_ values compared toTBA.

## Discussion

Data obtained from CD measurements clearly showed that the introduction of amino‐UNA, UNA or spacer‐C_3_ modifications in place of the thymidine nucleotide at positions T3, T7 and T12 was compatible with quadruplex formation, resulting in a similar structural topology to that of unmodified TBA. The appearance of an absorption minimum at 265 nm and a maximum at 295 nm has been reported as characteristic for an antiparallel single‐stranded G‐quadruplex topology and corresponds to the reported structure of TBA. Thus, all modified TBA sequences listed in Table 1 adapt the antiparallel chair‐like G‐quadruplex.

Next, UV melting studies were initiated to analyse the thermal stability of the TBA derivatives. Notably, UNA or spacer‐C_3_ substitution at T3, T7 or T12 increased the thermal denaturation temperature, whereas the temperature decreased in the case of amino‐UNA modifications. This could possibly be explained by the influence on the G‐quadruplex structure of the 2′–5′ internucleotide linkage between the sugar and the phosphate group or the 3′‐amino group. As amino‐UNA is a novel construct, there are no studies that can be used to explain this phenomenon directly. However, a recent study by Aher et al. investigated the impact of 2′–5′‐phosphodiester linkages and loop lengths on the folding topology of TBA.^21^ They showed that all variants with a 2′–5′‐linkage exhibited decreased melting temperatures. This effect was attributed to an increased number of bonds between the 5′‐O and 3′‐O‐phosphorus or/and an extended backbone geometry enabled by the 2′–5′ linkage. Even though amino‐UNA differs from the 3′‐deoxy‐2′–5′‐linked non‐genetic isoDNA studied by Aher et al., it seems plausible that the decreases in melting temperatures that we observed might be the result of a similar effect.

UNA and spacer‐C_3_ are less rigid than normal nucleotides and add more flexibility in the region in which they are introduced. The loop regions are known to make contributions to the stability of the G‐quadruplex due to π‐stacking between loop nucleobases and G‐quartets. Our data suggest that the increased flexibility of the loop regions might have led to the improved ability of loop nucleobases to interact with the G‐quartets, therefore resulting in enhanced thermal stability.^14^

For a better overview, the impact of modifying the TT loops and the TGT loop on thrombin clotting times are discussed separately:

### TT loops modification

Modification of the thymine residues at positions 3 and 12 lead to a reduction in the inhibitory effect, which was observed in the thrombin clotting experiments for all variants (amino‐UNA, spacer‐C_3_ and UNA). Krauss et al. showed that the two loops act as a pincer‐like system that binds the protruding region of thrombin exosite I.^10^, ^11^ Thus, the lack of thrombin inhibition of TBA‐SP3 and TBA‐SP12 is to be expected, as these oligonucleotides no longer possess the crucial thymines that facilitate the pincer formation. This is supported by our modelling results, which suggested that the substitution of either T3 or T12 with spacer‐C_3_ led to profound structural rearrangements, affecting the formation of a pincer‐like structure (Figure S2 D and F). Although they interact with the guanine tetrad, these modifications are no longer available for interactions with thrombin. This could explain not only the lack of inhibitory efficacy for both variants TBA‐SP3 and TBA‐SP12 but also the increase in thermal stability as π‐stacking interactions of either T3 or T12 with the guanine tetrads that could increase the stability of the structural core. For TBA‐A3 and TBA‐A12, as well as for TBA‐U3 and TBA‐U12, our modelling data suggested a similar change in structure (Figure S2 A, C, G and I). Even though the UNA and amino‐UNA variants still have the thymine residues at the modification site, it could be theorised that the flexibility of these nucleotides induce stabilisation of the G‐quadruplex through a higher stacking energy with the quartet. Consequently, this could result in a higher energy barrier to form the pincer‐like structure, which could negatively affect TBA and collectively account for its reduced activity towards thrombin.

In regard to the modification with amino‐UNA, additional explanations could be found. Recently reported crystal structures showed that residues in the TT loop interact with two hydrophobic clefts of the anion‐binding exosite I of thrombin (ABE1).^10^, ^11^ The substitutions of these loop residues (T3 and T12) with an amino‐UNA led to the introduction of a polar amino group. Thus, the hydrophobic interactions between the loop regions and thrombin could be reduced, resulting in a decreased inhibitory effect. Furthermore, reduction in the inhibitory ability of TBA‐A3, TBA‐A7 and TBA‐A12 could be the result of the observed lack of stability. Finally, results from the incorporation of UNA into TBA at positions T3 and T12 were in agreement with the work of Pasternak et al., who have previously reported that UNA incorporation at T7 (TBA‐U7) increased thrombin inhibition in anti‐thrombin assays by using blood plasma samples, whereas UNA incorporation at positions T3 and T12 (TBA‐U3 and TBA‐U12) reduced the effect.^14^

### TGT loop modification

The thrombin clotting time assay showed significantly prolonged clotting times for the variant TBA‐SP7, whereas TBA‐A7 and TBA‐U7 failed to show high thrombin inhibition. Only marginal interactions of thrombin with the TGT loop of TBA were reported by Krauss et al.^10^ Additionally, He et al. reported the relevance of the phosphate groups for the TBA–thrombin interaction.^22^ In regard to these reports, an explanation for the reduced inhibition could be found in the amino group of amino‐UNA, as this group could have a steric influence on these interactions in TBA‐A7. However, it was also suggested that the stability and rigidity of TBA is important for the interaction between aptamer and thrombin exosite I.^16^ As the variants with amino‐UNA exhibited the lowest melting temperatures of all tested variants, including TBA, this could also be an explanation for the reduced inhibitory effect of the amino‐UNA variants. In contrast, the spacer‐C_3_ variant is expected to exhibit the pincer‐like structure while also exhibiting increased stability; collectively this could account for the improved efficacy for TBA‐SP7 in the thrombin clotting time assay. It is worth mentioning that, for a direct comparison, the buffer condition must be the same in all above‐mentioned assay; however, we adopted the suggested buffer system reported by a previous group,^14^ which only allows for comparison between the unmodified TBA and the modified TBAs discussed here.

For TBA‐U7, our results deviate from the published data by Pasternak et al.^14^ They reported that UNA incorporation at T7 (TBA‐U7) increased the thrombin inhibition in anti‐thrombin assays using blood plasma samples. The different assays for measuring the effect of TBA derivatives on thrombin activity can be taken as a basis for this discrepancy. The thrombin clotting time assay measured the effect of TBA on thrombin in PBS, whereas the anti‐thrombin assay measured the activity in human plasma in which other plasma components could interact with thrombin and decrease the concentration available for thrombin interaction. Likewise, interactions with these other plasma components could destabilise the structure of TBA. Thus more stable forms, like TBA‐U7, could exhibit superior inhibitory activity in plasma, while being inferior in a buffer system like PBS. Another explanation for this discrepancy can be found in the different TBA concentrations used (50 nm of TBA‐U7 used in this study compared to 330 nm used by Pasternak et al.). Earlier studies have already shown that the concentration strongly affects the inhibitory capability of the TBA aptamer.^23^, ^24^ These discrepancies highlight the importance of testing modified aptamers in various settings in order to grasp possible drawbacks of prior in vivo and preclinical investigations.

Although electrostatic interactions contributed favourably to the overall binding of TBA, very highly unfavourable solvation energy compensated for the electrostatic energy, and as a result, nonpolar interactions such as vdW and lipophilic energies dominate the overall binding energy of these analogues (Figure S5). In general, amino‐UNA analogues bound with moderate affinity to thrombin (CT_50_ ranges from 45 to 55). From the binding affinity calculation, TBA‐SP3 (Δ*G*=−46.5 kcal mol^−1^) and TBA‐SP12 (Δ*G*=−57.7 kcal mol^−1^) should bind poorly to thrombin as compared to TBA‐SP7 (Δ*G*=−225.0 kcal mol^−1^), and this could be mainly due the loss of crucial interactions at the interface between the TBA‐SP3 analogue and residues at the thrombin active site, for example, Tyr117, Asn78 and Ile79 with T3 and Thr74, Arg75 and Tyr76 with T12. Moreover, nonpolar interactions (*E*_vdW_=−58.1 kcal mol^−1^) were significantly favourable for binding with thrombin (Table S1) as compared to rest of the analogues. It was clearly seen that TBA‐U7 binds significantly better than TBA‐U3 and TBA‐U12. Again, this might be due to the effect of interaction with thrombin residues. When positions T3 and T12 were modified with uUNA nucleotide, interactions between residues such as Thr74, Arg75, Tyr76, Asn78, Ile79 and Tyr117 with T3/T12 were lost. This is also significantly reflected in the overall binding affinity of U3 (Δ*G*=−33.5 kcal mol^−1^) and U12 (Δ*G*=−30 kcal mol^−1^) compared to U7 (Δ*G*=−65.7 kcal mol^−1^). We note from individual energy components contributions to the overall binding affinity that the value of *E*_vdW_ was quite low for U3 (−6.4 kcal mol^−1^) and U12 (−51.7 kcal mol^−1^) compared to that of U7 (−75.0 kcal mol^−1^).

In addition, we also performed combined molecular dynamics (to account for sampling effects at particular temperatures) and MM‐GBSA calculations to compute the binding free energy for TBA and TBA‐SP7 to thrombin. Both van de Waals and surface area energies in the binding energy clearly showed that nonpolar contribution is the main driving force to discriminate the TBA‐SP7 from TBA binding to thrombin (Figure 3). Energy decomposition analysis from free energy calculation examines the role of individual residue contributions to the overall binding affinity of the aptamers by decomposition of the binding free energy into aptamer–residue pairs. As seen from the decomposition analysis plot (Figure 3 E), TBA‐SP7 showed relatively different interaction patterns than TBA with thrombin residues during the simulation. For instance, residues Asp63, Arg75, Arg77_A, Asn78, Ile79 and Tyr117 showed a favourable energy contribution (≈2.00 Kcal mol^−1^ difference) to the binding affinity of TBA‐SP7 compared to TBA, especially Tyr117, which showed an additional energy contribution for TBA‐SP7 (−2.25 kcal mol^−1^) and whereas for TBA, this residue contributed very low (0.004 kcal mol^−1^). Moreover, some residues from the aptamer also equally contributed to the overall binding affinity of the complex as thrombin residues, for instance, T3, T4, G5, T12 and T13 resides. These aptamer–thrombin structural interaction observations are also in good agreement with Prime MM‐GBSA calculations. Analysis from the backbone RMSD and radius of gyration also suggested that the TBA‐SP7–thrombin complex was relatively more stable than the TBA–thrombin complex (Figure S6); in particular, when it was bound to thrombin, the overall RMSD remained less than 2.0 Å, whereas the thrombin–TBA complex was quite flexible throughout the simulation. In addition, the radius of gyration (of the Cα atoms) of aptamers (TBA and TBA‐SP7) from simulations was also computed and compared to the initial pose in order to provide a measure of overall compactness of molecular shape of aptamers.^25^, ^26^, ^27^ As seen from Figure S6 B, the overall molecular shape of TBA‐SP7 looks very similar to TBA when the aptamer binds to the thrombin. Another recent report^28^ showed the TBA structural stability and flexibility by using the static modes (SM) calculation including polythymines, PEG spacers and alkyl chain modifications in the TBA. We concluded that the introduction of spacers in the aptamer greatly affects not only the structural stability of TBA but also its ability to position itself within thrombin exosite I. Our observations, based on the MM‐GBSA calculations, also suggest that overall structural stability of aptamer–thrombin is not only governed by electrostatic energy (provides internal stability of aptamers) but also nonpolar interactions, especially van der Waals and surface area energy complementarity, which is essential for aptamers binding to the thrombin exosite I. This observation led us to believe that this is another demonstration of the malleability of G‐quadruplex‐forming aptamers, as previously observed for other aptamers.^28^, ^29^

## Conclusion

In summary, we have systematically investigated the importance of the flexibility of TBA loops by incorporating three different chemical modifications: spacer‐C_3_, amino‐UNA and UNA. All three modifications rendered high flexibility and less rigidity to TBA compared to natural nucleotide monomers, and this increased flexibility favoured stronger interactions of the nucleobase with the guanine tetrads, resulting in quadruplex formation with high thermal stability. In addition, the flexibility possibly favoured structural rearrangements of TBA‐type sequences, especially towards the pincer‐like structure of the TT loops and the orientation of T9. Unlike amino‐UNA‐ and UNA‐modified TBA sequences, one introduction of spacer‐C_3_ at position T7 of the TGT loop showed significant improvement in thermal stability and thrombin clotting time compared to unmodified TBA while maintaining the G‐quadruplex structure. We believe that these findings will be very useful in designing next‐generation thrombin‐binding aptamers.

## Experimental Section

**Scheme 3:**
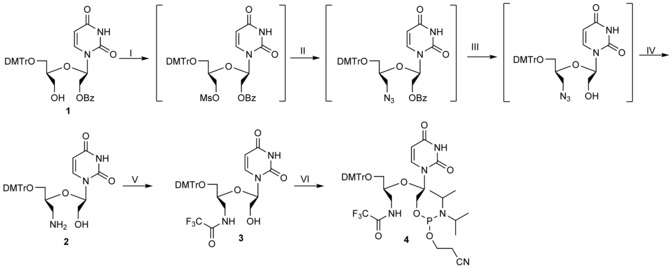
Synthesis of 3′‐amino‐UNA‐U nucleotide phosphoramidite. I) MsCl, anh. pyridine, 2 h, RT; II) NaN_3_, 15‐crown‐5, MeCN, 30 min, 130 °C in MW reactor; III) 0.1 m NaOH, MeOH, 16 h, RT; IV) Me_3_P, THF/H_2_O, 16 h, RT (62 % over four steps); V) ethyl trifluoroacetate, DMAP, MeOH, 2 h, RT (94 %); VI) 2‐cyanoethyl *N*,*N*,*N′*,*N′*‐tetraisopropyldiamidophosphite, diisopropylammonium tetrazolide, CH_2_Cl_2_, 5–16 h, RT (81 %).

**Oligonucleotide synthesis**: All oligonucleotides were synthesised by using standard phosphoramidite chemistry on an automated oligonucleotide synthesiser. All synthesised oligonucleotides were purified by ion exchange HPLC, and their composition was confirmed by MALDI‐TOF mass spectrometry. Synthesis of oligonucleotide sequences containing the 3′‐amino‐UNA‐U nucleotide monomer has not been reported elsewhere. Towards the construction of these novel oligonucleotide sequences with an oligonucleotide synthesiser, the synthesis methodology for 3′‐amino‐UNA‐U nucleoside phosphoramidite derivative (**4**, Scheme 3) is briefly described below.

Synthesis of amino‐UNA‐U phosphoramidite: Compound **1** (Scheme 3) was treated with methanesulfonyl chloride in pyridine to activate the alcohol. The crude mesylate was converted into an azide by dissolving it in acetonitrile and reacting it with sodium azide and 15‐crown‐5 ether in a microwave reactor. The benzoyl protecting group was removed by hydrolysis with sodium hydroxide in methanol. The final reduction of the deprotected azide was achieved under Staudinger conditions by employing trimethylphosphine as the reducing agent in a mixture of tetrahydrofuran and water, obtaining the desired 3′‐amino UNA‐U phosphoramidite in 62 % yield over four steps. The free amine was protected by using ethyl trifluoroacetate in MeOH with dimethylaminopyridine (DMAP) as a nucleophilic catalyst. The final amidite was furnished from 2‐cyanoethyl *N*,*N*,*N′,N′*‐tetraisopropyldiamidophosphite and diisopropylammonium tetrazolide in dichloromethane (detailed procedures are provided in the Supporting Information).

**Melting temperature studies**: Melting temperatures were recorded on a Beckman DU 800 spectrophotometer equipped with a six‐position microcell holder and a thermostat. Oligonucleotides (12.5 μm final concentration) were dissolved in buffer containing potassium chloride (100 mm) and sodium cacodylate (10 mm), pH 7. The samples were renatured for 10 min at 95 °C and then slowly cooled to room temperature. Absorbance versus temperature curves in 10 mm quartz microcuvettes were recorded at 295 nm. A temperature range of 15–85 °C was used at 0.5 °C min^−1^. Three spectra were recorded and averaged for each sample. The spectrum for buffer only (no sample added) was subtracted from each sample spectrum. Melting curves were analysed by using nonlinear curve fitting with the program MeltWin 3.5.^30^

**Circular dichroism (CD) spectra**: CD spectra were recorded on a Jasco J‐600A spectropolarimeter by using a 0.7 mL quartz cuvette with a 2 mm path length. Oligonucleotides (2.5 μm final concentration) were dissolved in phosphate buffer (10 mm, pH 6.9) containing KCl (5 mm). All samples were renatured for 10 min at 80 °C and then slowly cooled to room temperature prior to measurement at 15 °C with a 220–320 nm wavelength range. Five spectra were recorded and averaged for each sample. The buffer spectrum was subtracted from each sample spectrum.

**Thrombin clotting time assay**: Thrombin clotting times were measured spectrophotometrically on a PerkinElmer Lambda 35 UV/Vis spectrometer. Oligonucleotides were incubated for 1 min at 37 °C in PBS (1 mL) containing fibrinogen from human plasma (2 mg mL^−1^, F 3889, Sigma–Aldrich). Then, human thrombin (100 μL, 10 NIH per mL; T8885, Sigma–Aldrich) was added to the solution. The time required for fibrin polymerisation was determined from the UV scattering curve, which was registered as a function of time (wavelength fixed at 380 nm; total time: 600 s; time interval: 0.1 s) for each sequence. Each measurement was performed in triplicate at 50 nm concentrations of each oligonucleotide. The clotting time value, reported as average±standard deviation (AV.±S.D.) was derived from the midpoint of each scattering curve. This corresponded to the time at which 50 % of the final absorbance was observed.

**Computational modelling**

**Preparation of aptamers and protein**: The molecular structure of the human thrombin–aptamer (TBA) complex was obtained from the Protein Data Bank (PDB ID: 4DII, 2.06 Å resolution).^10^ The TBA was imported into the Maestro module available in the Schrödinger Suite (Schrödinger, LLC), and subsequently, the atomic coordinates of the aptamer were separated from the thrombin coordinates, and both were optimised separately. The thrombin structure was optimised by using the Protein Preparation Wizard.^31^ This protein structure optimisation includes adding hydrogen atoms, assigning bond orders and building disulfide bonds. The protonation states of the ionisable residues (pH 7) were predicted by the PROPKA tool provided in the Protein Preparation Wizard. An optimised structure model was finally found by energy minimisation (i.e., position of the hydrogen atoms) with the OPLS2005 force field.

For the aptamer, the protonation states were predicted by using the PROPKA tool in the presence of a K^+^ ion. Finally, the structure was energy‐minimised (only hydrogen atoms) by using the OPLS2005 force field. The structures of TBA derivatives (amino‐UNA, spacer‐C_3_ and UNA) used in these experiments were built manually by using the Maestro module available in the Schrödinger Suite from an optimised aptamer obtained from the X‐ray crystal structure.^10^ TBA derivatives were energy‐minimised to avoid any unfavourable contacts or steric clashes between the atoms. Subsequently, optimised structures were used for conformational analysis and MM‐GBSA‐based binding affinity prediction calculations.

**Conformational analysis**: In order to reveal the structural stability of various TBA derivatives, we initiated short stochastic dynamic simulations on these modified TBAs by using the Macro Model (version 9.1), as it is implemented in the Schrödinger Suite. All calculations were carried out by using the AMBER* force field^32^ with a GB/SA solvation model.^33^ In the process of thermalisation, initial velocities were generated from a Maxwell–Boltzmann distribution at 300 K. The SHAKE algorithm was utilised to constrain the lengths of all bonds involving hydrogen atoms. Coordinates were saved every 1.5 fs from the 0.5 ns simulation for the analysis. For minimisation, the PRCG (Polak–Ribiere Conjugate Gradient) protocol was applied with a convergence threshold of 0.05 kJ mol^−1^. Representative low‐energy structures for each TBA derivative were selected for further inspection.

**MM‐GBSA calculations**: In order to understand the binding affinity differences of various TBA complexes, MM‐GBSA calculations were initiated by using the Schrödinger Suite (Prime MM‐GBSA).^34^, ^35^ MM‐GBSA method implementation in the Schrödinger Suite is slightly different from the corresponding MM‐PBSA method described in our previous work.^29^ Whereas the MM‐PBSA method is based on molecular dynamics simulations, the Prime MM‐GBSA method calculates the binding affinity of the aptamer (Δ*G*_bind_) by energy minimisation procedures. The binding energy of the aptamer was extracted from an energy‐optimised thrombin–aptamer complex and Prime MM‐GBSA by using the VSGB 2.0 solvation (implicit) model.^33^ The Prime MM‐GBSA energy of the aptamer binding is estimated as [Eq. (1)]:

(1) $$\Delta G{}_{\mathrm{bind}}=E{}_{\mathrm{complex}}-E{}_{\mathrm{thrombin}}-E{}_{\mathrm{aptamer}}$$

This procedure is very efficient and is frequently used to rank protein–ligand complexes in the virtual screening process of lead identification/optimisation. During binding affinity prediction, the program offers the option to treat the aptamer and protein as flexible; in this study, a 5.0 Å region of the protein around the ligand was treated as flexible.

Extensive molecular dynamic simulations (eMD): Molecular dynamics (MD) simulations were carried out for the two chosen thrombin–aptamer complexes, namely thrombin:TBA (wild‐type) and thrombin:TBA‐SP7. The starting structure for the thrombin:TBA used in the MD simulation is based on the structure reported in the Protein Data Bank (PDB ID: 4DII).^10^ The starting structure for thrombin:TBA‐SP7 was obtained by modifying residue 7 (DT7) of the aptamer with a spacer group (‐CH_2_‐CH_2_‐CH_2_‐), and this structure was relaxed by using the AMBER force field.^36^ Both complexes were solvated in water and neutralised with a sufficient number of counter ions (seven Na^+^ ions). The K^+^ ion stabilising the aptamer geometry was also retained in both of the thrombin–aptamer complexes. The simulation box was chosen as orthorhombic and contained more than 20 000 water molecules. The force field for describing the aptamer and thrombin was FF99SB,^37^ as it contains parameters for both amino acids and nucleic acids, which are the fundamental units of proteins and DNA/aptamers, respectively. The water solvent was described by using the TIP3P force field. The space group parameters were based on general AMBER force field (GAFF), and the charges for this nonstandard residue were prepared based on fitting to the molecular electrostatic potential obtained by employing the Merz–Singh–Kollman Scheme.^38^ Structures were optimised with the HF/6‐31G* level of theory, as implemented in Gaussian09.^39^This protocol is often employed to describe any nonstandard residue or organic molecule in force field MD simulations. The solvated complexes were first energy‐minimised, then the simulations were carried out under constant temperature and pressure (room temperature, 1 atm pressure). The temperature was controlled by using a Langevin thermostat;^40^ the pressure was controlled by connecting the systems to a Berendsen's barostat.^41^ The time scale for integration of equation of motion was 2 fs. The initial timescale for the equilibration of the systems was 2 ns, and the total production run was 15 ns. The calculations were carried out by using Amber14 software.^42^The time evolution of total energies and system densities served as indicators for the equilibration runs. Further, the stability of both aptamer–thrombin complexes was verified by the potential energy landscape, root mean square displacement (RSMD) and radius of gyration. Free‐energy calculations (from 400 configurations), the RMSD and the radius of gyration analysis were performed for the last 5 ns trajectory of the production run. The MMPBSA.py tool^43^ of the Amber software was employed for this purpose. Various contributions to the total binding free energy, namely vdW, electrostatic, polar solvation and nonpolar solvation‐free energies were obtained, and the results are presented in Table ST2. The entropy calculations were also performed by using normal mode analysis (for 50 configurations collected at equal intervals from the last 2 ns run). The binding free energy analysis showed that the thrombin–TBA‐SP7 made a stronger complex than the thrombin–TBA (wild‐type) complex itself. The reason for this was analysed by using decomposition analysis, in which the individual residue contributions to total binding free energy can be computed.

## Conflict of interest

*The authors declare no conflict of interest*.

## Supporting information

As a service to our authors and readers, this journal provides supporting information supplied by the authors. Such materials are peer reviewed and may be re‐organized for online delivery, but are not copy‐edited or typeset. Technical support issues arising from supporting information (other than missing files) should be addressed to the authors.

Supplementary
