# Supplementary material for: Development of an Efficient G‐Quadruplex‐Stabilised Thrombin‐Binding Aptamer Containing a Three‐Carbon Spacer Molecule
Source: Chembiochem. 2017 Mar 15;18(8):755–63. doi: 10.1002/cbic.201600654 (PMC5413854; doi:10.1002/cbic.201600654)
Supplement: Supplementary file 1 — Supplementary [file CBIC-18-755-s001.pdf]

CHEMBIOCHEM

Supporting Information

Development of an Efficient G-Quadruplex-Stabilised Thrombin-Binding Aptamer Containing a Three-Carbon Spacer Molecule

Lukas J. Aaldering,[a, b] Vasanthanathan Poongavanam,[a] Niels Langkjær,[a] N. Arul Murugan,[c] Per Trolle Jørgensen,[a] Jesper Wengel,[a] and Rakesh N. Veedu*[a, d, e, f]

cbic_201600654_sm_miscellaneous_information.pdf

Author Contributions

L.J.A., V.P., N.A.M., N.L. and R.N.V. performed the experiments and computational modelling and wrote the manuscript. J.W., P.T.J. and R.N.V. conceived the idea and corrected the manuscripts.

Synthesis procedures for amino-UNA phosphoramidite (Figure 7, Main article text)

Compound **1 (Figure 7)** was treated with methanesulfonyl chloride in pyridine to activate the alcohol. The crude mesylate was converted in to an azide by dissolving it in acetonitrile and reacting it with sodium azide and 15-crown-5 ether in a microwave reactor. The benzoyl protecting group was removed by hydrolysis with sodium hydroxide in methanol. The final reduction of the deprotected azide was achieved using Staudinger condition by employing trimethylphosphine as reducing agent in a mixture of tetrahydrofuran and water obtaining the desired 3'-amino UNA-U phosphoramidite in 62% over four steps. The free amine was protected using ethyl trifluoroacetate in methanol with dimethylaminopyridine (DMAP) as a nucleophilic catalyst. The final amidite was furnished from 2-cyanoethyl *N*,*N*,*N'*,*N'*-tetraisopropyldiamidophosphite and diisopropylammonium tetrazolide in dichloromethane (detailed procedures are provided in the supplementary information).

Experimentals:

3'-deoxy-5'-*O*-(4,4'-dimethoxytrityl)-3'-amino-2',3'-secouridine (2): 1 (2.81 g, 4.31 mmol) was dissolved in anh. pyridine (20 mL) and MsCl (0.50 mL, 6.46 mmol) was added and allowed to stir for 2 h at rt. EtOH (5 mL) was added and the mixture was stirred for 10 min and then poured into DCM (50 mL) and washed with sat. aq. $NaHCO_3$ (2 × 75 mL). The organics were pooled and dried over $MgSO_4$, then coevaporated using toluene to give the crude mesylate. The crude mesylate was dissolved in anh. MeCN (13 mL) and transferred to a microwave tube along with NaN_3 (845 mg, 13.0 mmol) and 15-crown-5 (2.60 mL, 13.1 mmol). The tube was sealed and heated to 130 °C for 30 min. The reaction mixture was poured into DCM (100 mL) and washed with brine (100 mL) and H_2O (100 mL). The organics were collected and dried over $MgSO_4$ followed by filtration and evaporation to yield the crude azide.

The crude azide was then dissolved in MeOH (75 mL), and to this was added NaOH (919 mg) in MeOH (150 mL). The mixture was allowed to stir overnight at rt. Sat. aq. NH_4Cl (25 mL) was added and the reaction mixture was reduced to approximately 50 mL, then poured into DCM (100 mL) and washed with H_2O (3 × 100 mL). The organics were pooled and dried over $MgSO_4$ followed by filtration and evaporation under reduced pressure.

The crude 3'-azido UNA U was dissolved in THF (60 mL) along with H_2O (10 mL), and Me_3P in THF (1 M, 22.0 mL, 22.0 mmol) was added. The mixture was allowed to stir

overnight at rt. THF was removed under reduced pressure and the crude was redissolved in DCM (100 mL). The crude mixture was washed with H_2O (2 × 200 mL) and brine (100 mL). The organics were evaporated and the crude 3'-amino UNA U was purified using an automated flash chromatography using a biotage SP4 instrument giving **2** as 1.48 g of white foam (62 % over 4 steps). ^{1}H NMR (400 MHz, DMSO) δ 7.60 (d, J = 8.0 Hz, 1H, H5), 7.39 – 7.07 (m, 9H, DMTr), 6.86 (d, J = 8.3 Hz, 4H, DMTr), 6.50 – 4.00 (bs, 1H,), 5.75 (dd, J = 6.1, 5.2 Hz, 1H, H1'), 5.51 (d, J = 8.0 Hz, 1H, H6), 3.73 (s, 6H, 2 × OMe), 3.69 – 3.50 (m, 3H, H2'/H4'), 3.45 - 3.10 (bs, 1H), 2.97 (ddd, J = 14.1, 10.2, 5.3 Hz, 2H, H5'), 2.65 (ddd, J = 19.6, 13.3, 5.4 Hz, 2H, H3'). ^{13}C NMR (101 MHz, DMSO) δ 163.2, 158.0, 151.1, 144.8, 141.0 (C5), 135.6, 135.5, 129.6 (DMTr), 129.5 (DMTr), 127.7 (DMTr), 127.6 (DMTr), 126.6 (DMTr), 113.1, 101.6 (C6), 85.4 (DMTr), 84.2 (H1'), 80.2 (H4'), 69.8, 64.0 (H5'), 61.7 (H2'), 55.0 (2 × OMe), 42.4 (H3'). ESI-HRMS ($M+Na^+$): *m/z* 570.2204 Calc.: 570.2211. Elementary analysis for $C_{30}H_{33}N_3O_7$•1.25 H_2O: C, 63.20; H, 6.28; N, 7.37. Found: C, 63.30; H, 5.93; N, 7.44.

3'-deoxy-5'-*O*-(4,4'-dimethoxytrityl)-3'-trifluoracetamide-2',3'-secouridine (3): Nucleoside **2** (200 mg, 0.037 mmol) was dissolved in MeOH (5 mL) along with DMAP (46 mg, 0.037 mmol). The mixture was added ethyl trifluoroacetate (0.27 mL, 2.47 mmol) and stirred at rt for 2 h. MeOH was evaporated and the residue was purified using an automated flash chromatography using a biotage SP4 instrumentgiving **3** as a white solid (220 mg, 94 %). ^{1}H NMR (400 MHz, DMSO) δ 11.33 (s, 1H, NH), 9.40 (s, 1H, NHC=O), 7.58 (d, *J* = 8.0 Hz, 1H, H5), 7.40 – 7.09 (m, 9H, DMTr), 6.91 – 6.80 (m, 4H, DMTr), 5.72 (t, *J* = 5.8 Hz, 1H, H1'), 5.49 (d, *J* = 8.0 Hz, 1H, H6), 5.16 (s, 1H, 2'-OH), 3.76 – 3.67 (m, 7H, 2 × OMe/H4'), 3.67 – 3.48 (m, 2H, H3'), 3.42 – 3.22 (m, 2H, H3'), 3.07 – 2.86 (m, 2H, H5'). ^{19}F NMR (376 MHz, DMSO) δ -74.36. ^{13}C NMR (101 MHz, DMSO) δ 163.1, 158.0, 156.7, 151.3, 144.6, 140.9, 135.4, 135.3, 129.5, 127.8, 127.6, 126.7, 113.1, 101.8, 85.7, 83.8, 76.7, 63.7, 61.0, 55.0, 54.9. ESI-HRMS ($M+Na^+$): *m/z* 666.2046, calc. 666.2034. Elementary analysis: $C_{32}H_{32}F_3N_3O_8$•0.5 eq. H_2O: C, 59.30; H, 5.05; N, 6.48. Found: C, 59.39; H, 4.90; N, 6.48.

2'-*O*-(2-cyanoethoxy(diisopropylamino)phosphino)-3'-deoxy-5'-*O*-(4,4'-dimethoxytrityl)-3'-trifluoracetamide-2',3'-secouridine (4): Nucleoside 3 (213 mg, 0.033 mmol) was dissolved in anh. DCM (5 mL) together with diisopropylammonium tetrazolide (85 mg, 0.05 mmol). To the solution was added 2-cyanoethyl *N*,*N*,*N'*,*N'*-tetraisopropyldiamidophosphite (0.16 mL, 0.05 mmol) and the reaction was left overnight at rt. The reaction was washed with sat. aq. $NaHCO_3$ (3 × 10 mL), followed by drying over $MgSO_4$, filtration and evaporation of the organics. 4 were triturated from PE and EtOAc to

give a white solid (225 mg, 81 %). ^{31}P NMR (162 MHz, $CDCl_3$) δ 149.02, 148.70, 148.63, 148.56. ^{19}F NMR (376 MHz, $CDCl_3$) δ -67.33, -67.38, -68.53, -68.58. ESI-HRMS (M+Na^+): *m*/*z* 866.3092, calc.: 866.3112.

Figure S1. UV melting analysis of TBA and TBA-variants

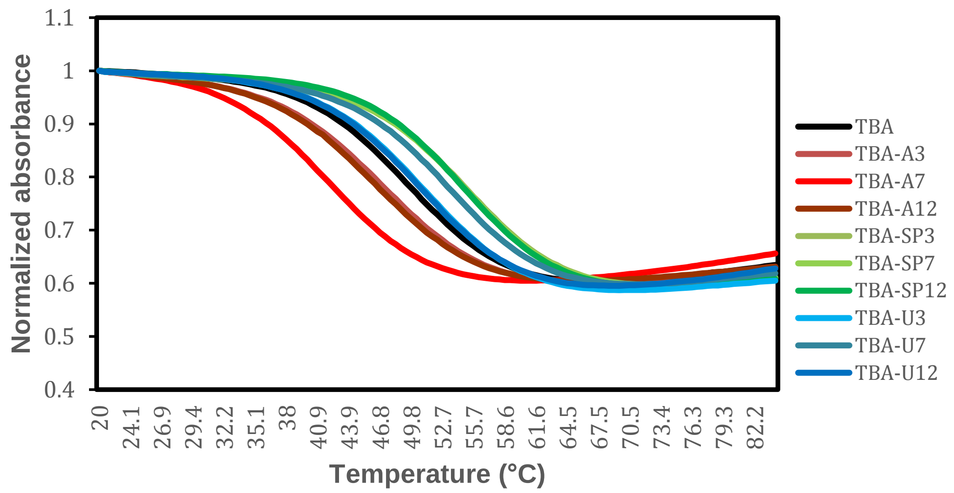

Figure S2. Biolayer interferometry-based binding analysis of TBA and TBA-SP7.

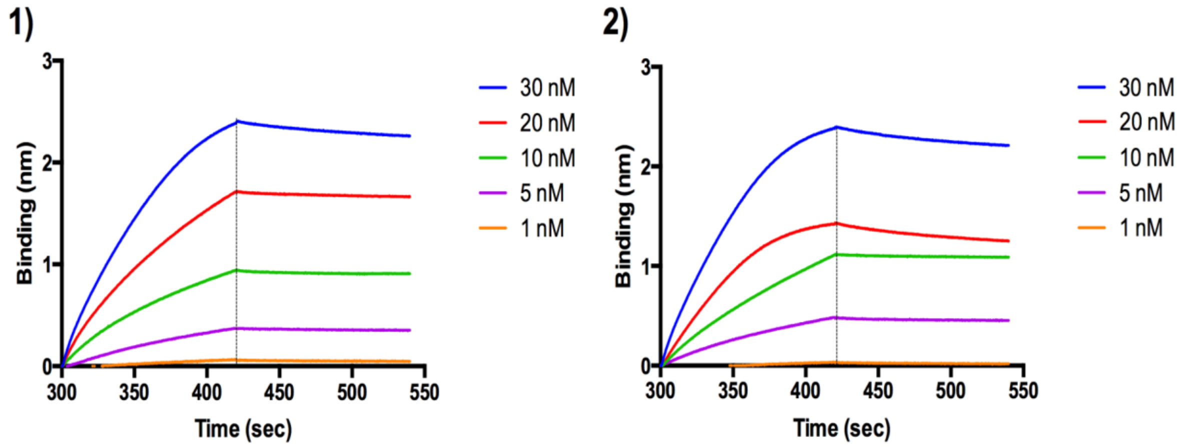

Figure S3. Binding pose of TBA derivatives is shown. The position at which a T nucleotide is modified with a UNA (A = TBA-A3, B = TBA-A7, C = TBA-A12), a Spacer-C3 (D = TBA-SP3, E = TBA-SP7, F = TBA-SP12) and an aUNA (G = TBA-U3, H = TBA-U7, I = TBA-U12) is indicated with green color.

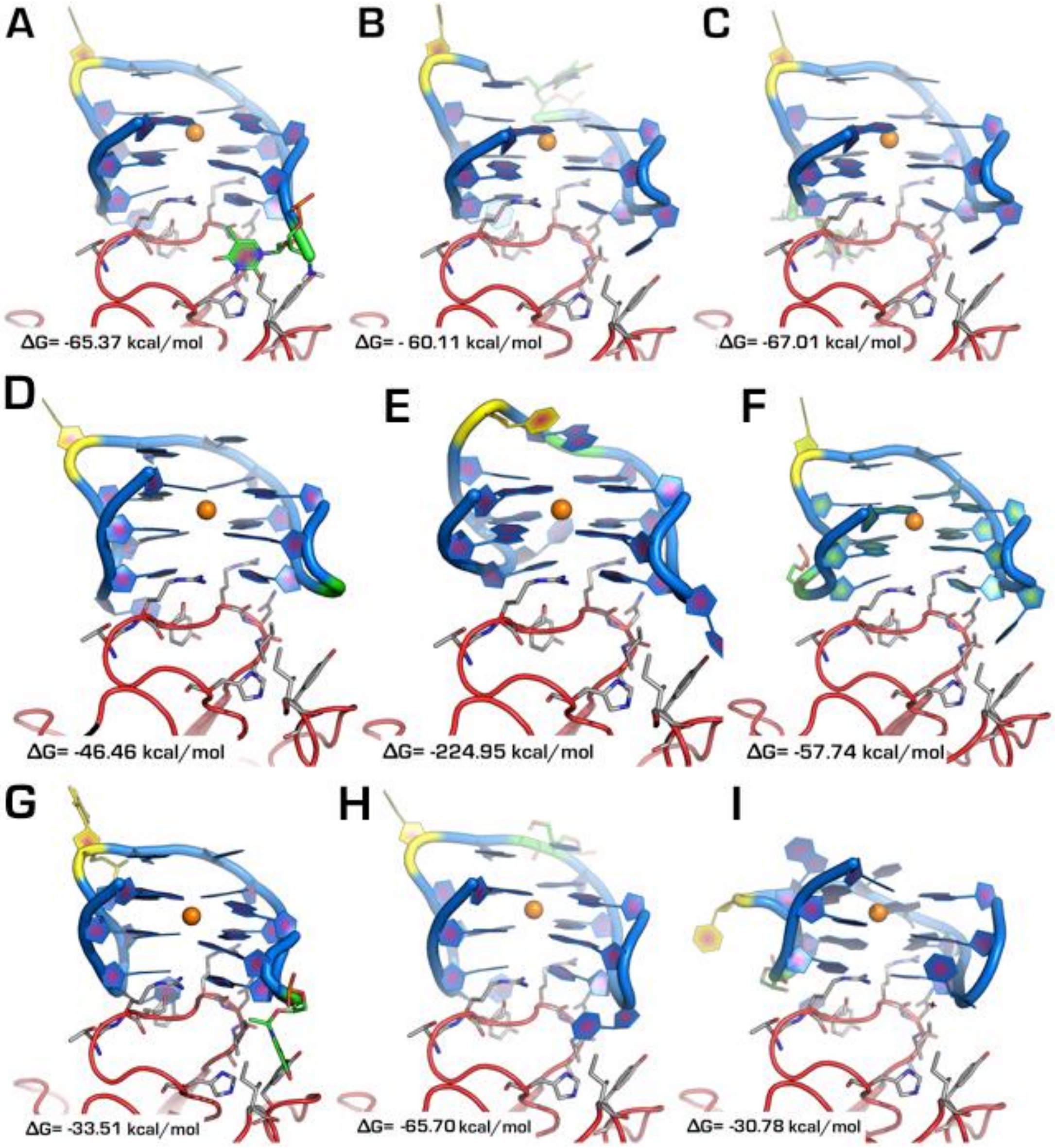

Figure S4. A. Correlation between the observed (Fibrinogen clotting times), B. estimated binding affinities (ΔG_{bind}) using the MM-GBSA calculations, here, red and green bar corresponds to the binding affinity (ΔG_{bind}) and the CT_{50} activity, respectively.

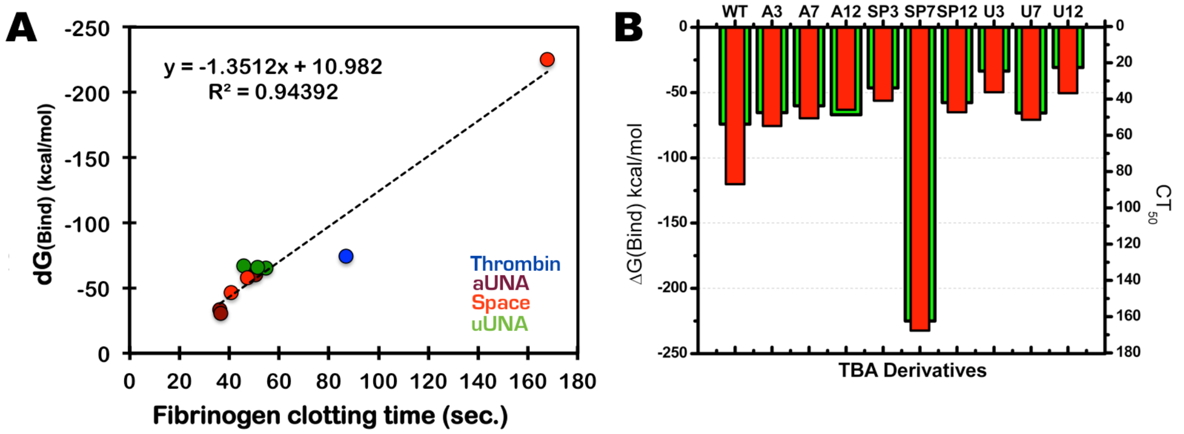

Figure S5: A. Comparison of covalent energy and covalent binding energy of different aptamers; B: relationship between CT50 and MM-GBSA energy (excluding $dG_{covelent}$).

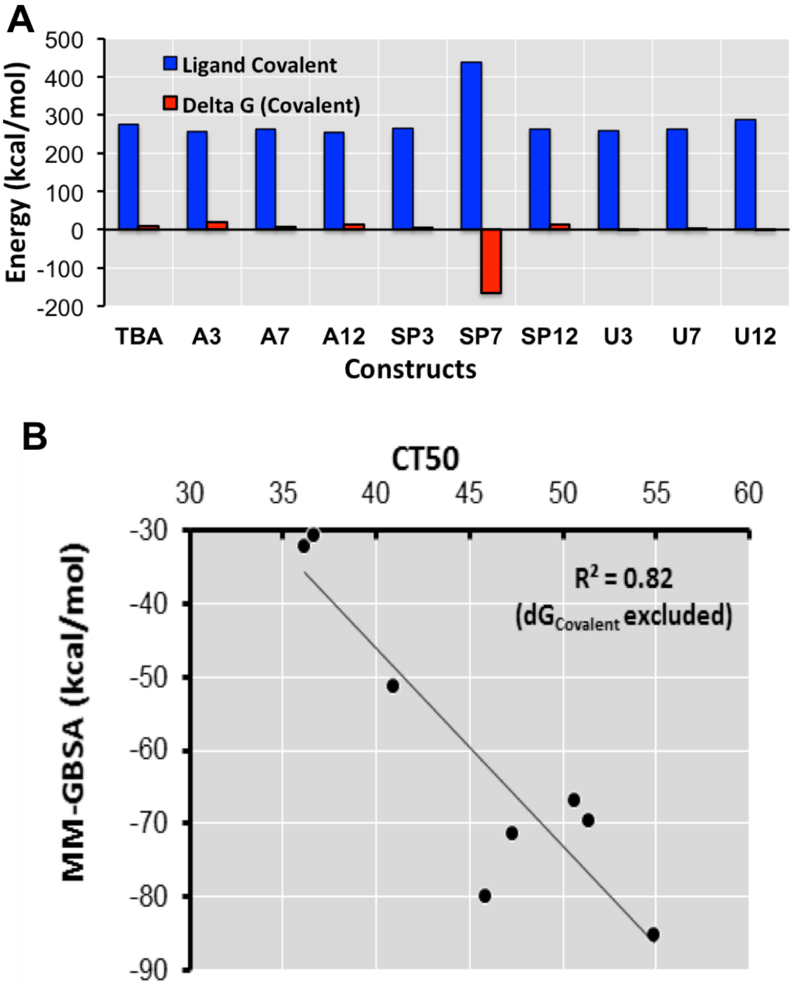

Figure S6: Comparison of various molecular surface area of various TBA analogues (TBA, TBA-SP7 and TBA-U3), colour representing according to the energy contributions to total binding affinity (MM-GBSA (ΔG) of each aptamers in the thrombin. Energy components such as $E_{electrostatic}$ (A), $E_{GB\text{-}solvation}$ (B), $E_{van\ der\ Waals}$

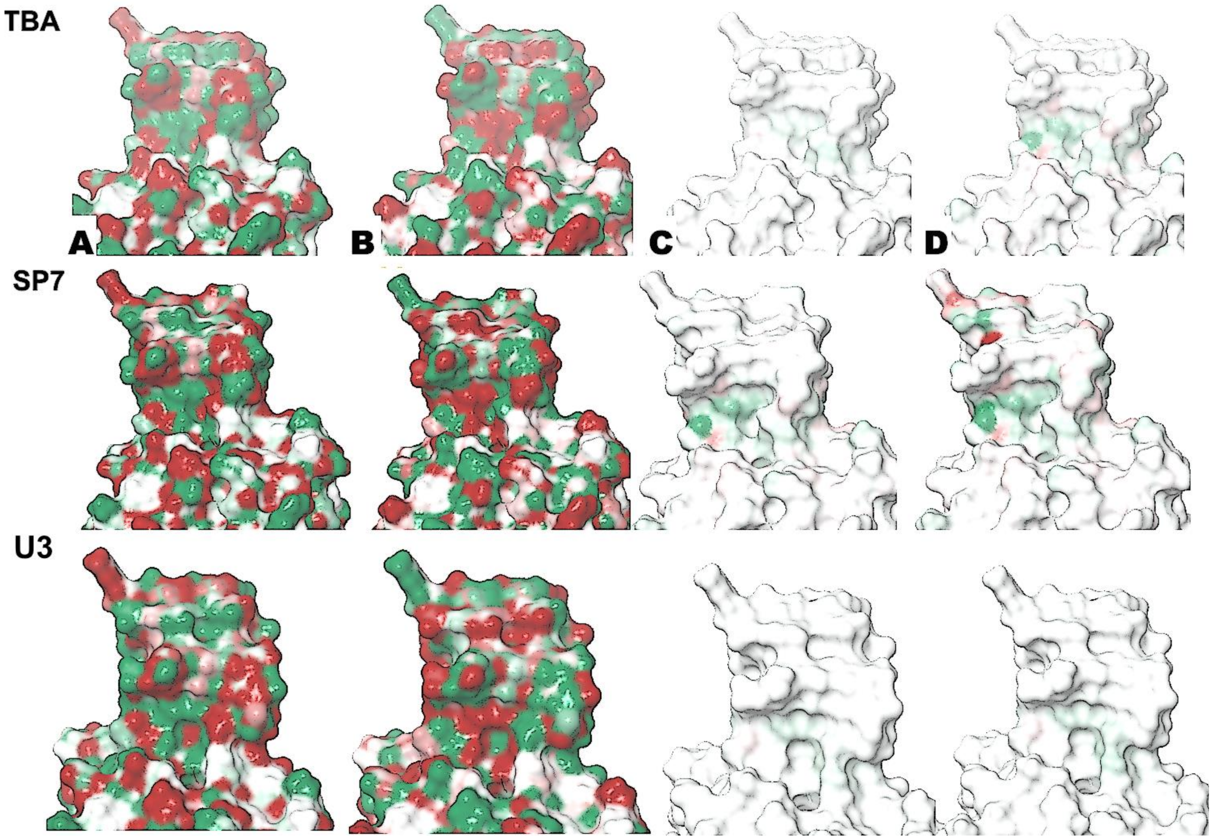

Figure S7: Comparison of backbone RMSD and Radius of Gyration for TBA and TBA-SP7. complex (a) and Aptamer (b) are highlighted. Conformations from the last 5 ns of 15 ns MD simulation were compared to initial structure.

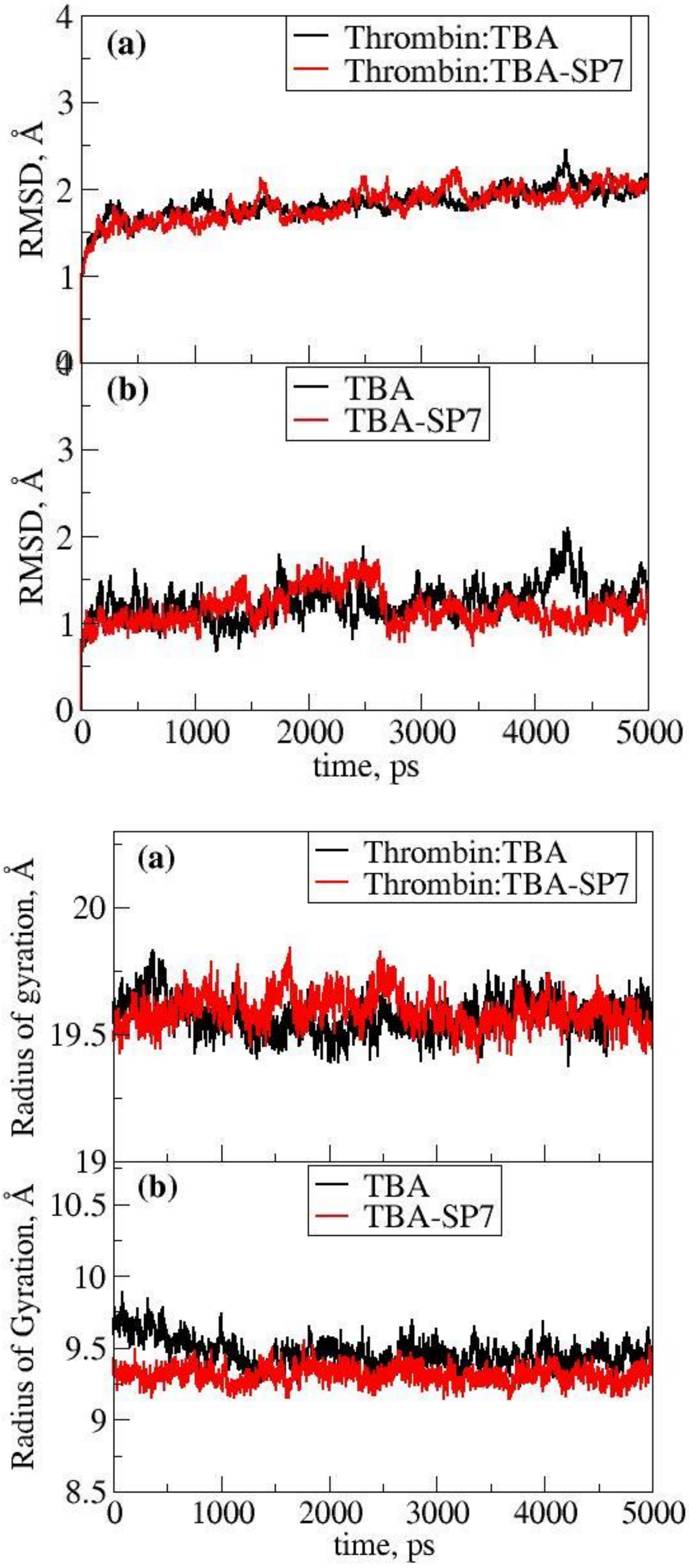

Table ST1: Summary of energies obtained from MM-GBSA method. All units are reported in kcal/mol

	**Compt.**	**TBA-Wildtype**	**TBA_A3**	**TBA_A7**	**TBA_A12**	**TBA_SP3**	**TBA_SP7**	**TBA_SP12**	**TBA_U3**	**TBA_u7**	**TBA_U12**
Complex	Coul	-9504.01	-9564.25	-9869.41	-9917.07	-9406.05	-9473.13	-9453.15	-8907.09	-9584.12	-9774.92
	Covalent	2409.62	2399.8	1766.05	1764.12	2394.69	2402.3	2387.8	2386.13	2386.07	1784.78
	Hbond	-136.15	-135.77	-160.21	-160.21	-135.5	-136.15	-136.45	-136.15	-136.47	-160.21
	Lipo	-1634.96	-1649.66	-1649.25	-1653.74	-1635.44	-1634.96	-1635.57	-1639.02	-1636.28	-1651.14
	Packing	-10.45	-8.78	-11.53	-12.59	-9.11	-10.45	-8.77	-10.45	-8.75	-11.54
	SelfCont	-114.39	-114.58	-108.8	-108.8	-114.57	-114.39	-114.5	-114.39	-115.52	-108.8
	Solv GB	-3614.71	-3516.39	-3486.62	-3441.18	-3569.55	-3495.52	-3525.45	-4181.06	-3468.35	-3533.6
	vdW	-319.64	-322.57	-1485.83	-1484.16	-303.71	-313.21	-321.4	-260.77	-317.76	-1491.88
	**Sum**	**-12924.7**	**-12912.2**	**-15005.62**	**-15013.65**	**-12779.24**	**-12775.51**	**-12807.47**	**-12862.8**	**-12881.19**	**-14947.3**
Receptor	Coul	-8180.66	-8180.71	-8415.31	-8415.31	-8180.71	-8185.15	-8174.04	-8185.15	-8181.29	-8415.31
	Covalent	2124.59	2124.61	1496.8	1496.8	2124.61	2129.59	2112.45	2129.59	2120.22	1496.8
	Hbond	-136.68	-136.68	-160.21	-160.21	-136.68	-136.15	-137.08	-136.15	-136.48	-160.21
	Lipo	-1636.36	-1636.35	-1646.49	-1646.49	-1636.35	-1634.96	-1636.68	-1634.96	-1634.66	-1646.49
	Packing	-8.68	-8.68	-11.53	-11.53	-8.68	-10.45	-9.07	-10.45	-8.74	-11.53
	SelfCont	-113.47	-113.47	-108.8	-108.8	-113.47	-114.39	-113.43	-114.39	-115.52	-108.8
	Solv GB	-2465.74	-2465.78	-2493.42	-2493.42	-2465.78	-2461.48	-2465.69	-2461.48	-2459.29	-2493.42
	vdW	-147.23	-147.23	-1319.97	-1319.97	-147.23	-146.96	-157.58	-146.96	-148.76	-1319.97
	**Sum**	**-10564.24**	**-10564.3**	**-12658.93**	**-12658.93**	**-10564.3**	**-10559.95**	**-10581.11**	**-10559.95**	**-10564.51**	**-12658.93**
Ligand	Coul	384.53	196.03	183.42	136.71	306.63	304.89	287.26	228.06	200.34	328.9
	Covalent	274.97	255.42	262.56	254.24	265.13	438.07	261.8	257.78	261.98	288.18
	Hbond	0	0	0	0	0	0	0	0	0	0
	Lipo	0	-2.62	-2.75	-2.76	0	0	0	-2.75	-1.63	-1.87
	Packing	0	0	0	0	0	0	0	0	0	0
	SelfCont	0	0	0	0	0	0	0	0	0	0
	Solv GB	-2847.53	-2627.35	-2632.06	-2576.77	-2634.94	-2625.4	-2610.28	-2645	-2617.71	-2752.6
	vdW	-98.19	-104.01	-97.75	-99.13	-105.29	-108.16	-107.39	-107.43	-93.95	-120.19
	**Sum**	**-2286.23**	**-2282.52**	**-2286.58**	**-2287.7**	**-2168.48**	**-1990.6**	**-2168.61**	**-2269.33**	**-2250.97**	**-2257.58**
Complex-Receptor-Ligand	Coul	**-1707.87**	**-1579.58**	**-1637.53**	**-1638.48**	**-1531.97**	**-1592.88**	**-1566.38**	**-950**	**-1603.17**	**-1688.51**
	Covalent	**10.06**	**19.77**	**6.68**	**13.07**	**4.95**	**-165.36**	**13.56**	**-1.24**	**3.87**	**-0.2**
	Hbond	**0.53**	**0.91**	**0**	**0**	**1.18**	**0**	**0.63**	**0**	**0**	**0**
	Lipo	**1.4**	**-10.68**	**-0.01**	**-4.49**	**0.91**	**0**	**1.12**	**-1.31**	**0**	**-2.78**
	Packing	**-1.77**	**-0.1**	**0**	**-1.05**	**-0.43**	**0**	**0.3**	**0**	**-0.01**	**0**
	SelfCont	**-0.91**	**-1.11**	**0**	**0**	**-1.09**	**0**	**-1.07**	**0**	**0**	**0**
	Solv GB	**1698.55**	**1576.74**	**1638.86**	**1629**	**1531.17**	**1591.36**	**1550.53**	**925.42**	**1608.65**	**1712.42**
	vdW	**-74.22**	**-71.33**	**-68.12**	**-65.07**	**-51.19**	**-58.08**	**-56.43**	**-6.38**	**-75.05**	**-51.72**
	**DG**	**-74.24**	**-65.38**	**-60.12**	**-67.02**	**-46.46**	**-224.96**	**-57.74**	**-33.51**	**-65.7**	**-30.79**

Note: All energy components are extracted from the differences of $\Delta G_{Complex}$- ΔG_{Ligand}- $\Delta G_{Receptor.}$ All units are reported in kcal/mol. Abbreviation: E_{Coul} = Coulomb energy, $E_{Covalent}$ = Covalent binding energy, E_{vdW} = Van der Waals energy, E_{Lipo} = Lipophilic energy, $E_{GB(Solv)}$ = Generalized Born electrostatic solvation energy, E_{Hbond} = Hydrogen-bonding energy, $E_{Packing}$ = Packing Pi-pi packing energy, $E_{SelfCont}$ = Self-contact correction.

ST2: Summary of (Binding) Energies Obtained from Final MM-GBSA

	TBA-SP7			TBA		
Energy	Average	Std.	Err.	Average	Std.	Err.
VDWAALS	-65.6948	4.066	0.2033	-58.5707	4.2886	0.2144
EEL	-1234.2791	39.4407	1.972	-1151.3057	38.7173	1.9359
EGB	1250.8703	38.952	1.9476	1179.426	38.3451	1.9173
ESURF	-8.0951	0.2461	0.0123	-7.8191	0.5781	0.0289
DELTA_Gas	-1299.9739	39.716	1.9858	-1209.8764	40.3772	2.0189
DELTA_Sol	1242.7752	38.9428	1.9471	1171.607	38.0906	1.9045
DELTA G	-57.1987	4.7514	0.2376	-38.2694	6.4184	0.3209

Std: standard deviation and Err: Error
